# Fat Versus Carbohydrate-Based Energy-Restricted Diets for Weight Loss in Patients With Type 2 Diabetes

**DOI:** 10.1007/s11892-018-1103-4

**Published:** 2018-10-17

**Authors:** Osama Hamdy, Mhd Wael Tasabehji, Taha Elseaidy, Shaheen Tomah, Sahar Ashrafzadeh, Adham Mottalib

**Affiliations:** 1 Joslin Diabetes Center, Harvard Medical School, One Joslin Place, Boston, MA 02215 USA; 2 Department of Medicine, Lahey Hospital and Medical Center, 41 Mall Rd, Burlington, MA 01805 USA

**Keywords:** Type 2 diabetes, Obesity, Weight management, Low-carbohydrate diet, High-carbohydrate diet, High-fat diet, Ketogenic diet

## Abstract

**Purpose of Review:**

The prevalence of combined obesity and diabetes has increased dramatically in the last few decades. Although medical and surgical weight management are variably effective in addressing this epidemic, it is essential to parallel these strategies with a hypocaloric diet comprising the appropriate macronutrient composition to induce weight loss, enhance glycemic control, and improve cardiovascular risk factors. This review reports the current evidence of the role of carbohydrates and fat-based diets for weight management in patients with combined type 2 diabetes (T2D) and obesity.

**Recent Findings:**

Low-carbohydrate diets were shown to decrease postprandial glucose levels whereas high-carbohydrate, low-fat diets are considered cardio-protective.

**Summary:**

A diet with an optimal macronutrient composition remains uncertain for patients with combined T2D and obesity. Further research is still needed to define the best dietary composition that achieves the maximum benefits on weight management, glycemic control, and cardiovascular risk factors.

## Introduction

Diabetes mellitus (DM) has become a huge burden on healthcare worldwide. Recent studies estimated that almost 30 million Americans (approximately 9.4% of the US population) were diagnosed with DM in 2015 [1]. This percentage reached 25.2% among adults who are 65 years or older [1], demonstrating the need for extensive effort to reduce its burden from individual and healthcare perspectives. There is strong evidence of an association between obesity and type 2 diabetes mellitus (T2D) [2]. A recent report estimates that almost 87.5% of patients with T2D are either overweight or obese [1]. Healthy overweight or obese people are at a higher risk for developing T2D compared to people who fall within the normal body mass index (BMI) range [3, 4]. In addition, obesity is a major risk factor for developing hypertension, cardiovascular diseases (CVD), and strokes. These risks are much higher when obesity is accompanied by T2D [5].

The pertinent and enlarging need for more efficient strategies to manage T2D is due to its continuous surge in prevalence despite the recent advances in its pharmacotherapy [6**•**]. Among the various risk factors for developing T2D, poor diet, decreased physical activity, and obesity stand out [7]. Poor diet with a high amount of sugar and greater intake of finely processed grains and starchy carbohydrates is associated with T2D development [8].

While poor diet is considered a major risk factor for developing T2D, nutrition therapy (NT) using optimal diet may effectively control body weight and hyperglycemia in patients with T2D. The current recommendation of the American Diabetes Association (ADA) states that for weight loss in patients with T2D, either low-carbohydrate, low-fat calorie-restricted, or Mediterranean diet may be effective NT and that the mix of carbohydrate, protein, and fat may be adjusted [9]. In the same recommendation, it was mentioned that at least 150 g of carbohydrates daily is suggested for people diagnosed with T2D [9].

In fact, there is a notable debate regarding nutrition intervention for weight management in patients with combined T2D and obesity, and the proper diet for effective and long-lasting weight management is not yet identified. Researchers in the Look AHEAD study used hypocaloric diets lower in fat. Despite many improvements achieved with weight loss in the study, including A1C and lipid profile, the study findings indicated no relationship between weight loss and cardiovascular outcomes in patients with combined T2D and obesity [2]. While further research on the effects of weight loss on CVD risk is warranted, the Look AHEAD study shed light on the importance of weight loss in managing combined T2D and obesity due to reduction in the number of medications, reduced hospitalization rates, and reduced incidence of chronic kidney disease and depression [2].

## Nutrition Therapy

Nutrition therapy is an essential aspect of diabetes management. Improving energy intake and macronutrient composition are the main items of the current research of NT [10]. It is known that calorie restriction is vital in achieving both glycemic control and preferable lipid profiles. However, optimal macronutrient composition for patients with combined T2D and obesity is still unclear. Some studies show that a Mediterranean diet has superior effects on weight loss compared to a low-fat diet [11, 12]. Sacks et al. concluded that clinically significant weight loss can be achieved through calorie-reduced diets regardless of macronutrient composition [13], however their study demonstrated a common overlap between behavioral elements of eating and macronutrient compositions where most of their studied subjects ended up losing nearly the same amount of weight by the end of the study period (2 years) with dissolved differences in macronutrient compositions. If we consider this behavioral element, the study did not help in answering the important question: which macronutrient composition is superior for sustained weight reduction?

While initial weight loss is faster when utilizing a low-carbohydrate (LC) diet in comparison to a low-fat (LF) diet, the long-term weight loss at 1 year is nearly similar with no superiority of either diet in terms of changes in A1C or blood pressure with only a change in high-density lipoprotein (HDL), which increased in the LC group [14]. In patients with T2D, LC diets may have specific benefits on glycemic parameters. Carbohydrates are the main source of glucose for metabolism, and reducing their intake may lead to a decrease in insulin requirements and an improvement in insulin sensitivity that results in reduction of postprandial hyperglycemia [15, 16]. However, most of these studies are limited by their small sample size, lack of control groups, or short follow-up periods [17]. While a preferred LC diet is composed of carbohydrates with low glycemic index (GI), the amount of these carbohydrates is still uncertain [18]. Diets containing similar amounts of simple sugars and different glycemic indexes did not show an association between high GI and the chance of developing insulin resistance among non-diabetic study subject [19].

## Low-Carbohydrate Diet

LC diets have become popular due to their ability to induce rapid weight loss. Examples include the Zone diet, South Beach diet, Atkins diet, and other ketogenic diets [20]. Some suggest LC diets as the first choice in managing T2D [21]. However, the definition of LC diet varies broadly. A recent meta-analysis defined it as a diet with a total energy intake (TEI) from carbohydrates of less than 45%. Others recommend lower amounts of carbohydrates and even support a very low carbohydrate ketogenic diet (VLCKD) of less than 50 g of carbohydrates per day (10% of TEI for a 2000 Kcal diet) [15]. Some observational studies revealed that a higher percentage of TEI from carbohydrates might play a role in increasing overall caloric intake, which itself leads to obesity and increased BMI [22]. In contrast, other large observational studies suggested exactly the opposite [23].

Prior studies also limited glycemic control assessment to two factors only: A1C and fasting plasma glucose [14, 24–26]. However, glycemic variability (GV; amplitude, frequency, and duration of diurnal glucose fluctuations) and Post Prandial Glucose (PPG) excursions are also considered independent risk factors for diabetes complications, including CVD risk [27, 28]. Only one study was designed for this purpose and evaluated the effect of a diet (composed of LC content, high unsaturated fat and low saturated fat) on glycemic control and CVD risk factors. Participants in that study were overweight or obese patients with T2D [6**•**]. Both LC and high-carbohydrate (HC) diets had approximately comparable effects on weight loss and glycemic control, but LC diet was superior in stabilizing diurnal blood glucose and lipid profile [6**•**].

LC diets improve glycemic control and hyperinsulinemia in patients with T2D [29]. Additionally, the lower insulin secretion caused by LC diets lead to increased lipolysis, increased fatty acid oxidation, and reduced lipogenesis [30]. Fasting lipids usually improve with LC diets but are dependent on the quality and type of dietary fats utilized to replace carbohydrates as well as the total amount of carbohydrates. However, one of the possible concerns of LC with high-fat diets is postprandial hyperlipidemia [29].

Advocates of LC diets created the term “metabolic advantage,” stating that when these diets are utilized for weight loss, energy expenditure remains elevated [31]. However, in a study by Hall et al., carbohydrate restriction resulted in a decrease of energy expenditure (about 98 Kcal/day), while iso-caloric diet with lower amounts of fats did not lead to such an outcome [32].

It is claimed that LC diets might result in increased weight loss by their capacity to decrease calorie intake by suppressing appetite. This is mostly due to increased amounts of circulating ketones that play a role in suppressing appetite [33] and possible consumption of higher protein in replacement of reduced carbohydrates, which plays a similar role in increasing satiety [33].

On body weight, high-fat–LC diets and low-fat–HC diets were shown to have similar effects on body weight, blood pressure, and insulin concentrations [14, 24, 25], but LC diets have greater impact in improving glycemic control [24–26, 34]. However, when fat type (reduced saturated fat) is matched between HC and LC diets, both resulted in substantial improvement in glycemic control and several CV risk factors [35].

In a very recent prospective cohort study for 25 years exploring association between carbohydrate consumption and mortality, both LC (< 40%: from vegetables, fruits, and grains) and HC (> 70%: from refined carbohydrates such as white rice) diets were linked to increased mortality among people without diabetes [36**••**]. Meanwhile, diets composed of 50–55% carbohydrates (regardless their plants or animal source) were associated with lowest risk of mortality. When comparing LC diets, higher mortality rates were associated with LC diets with animal-based protein and fats, while lower mortality rates were noticed among individuals who consumed LC diets with plant-based protein and fats. This suggests that food source plays an important role in modifying the linkage between carbohydrate intake and mortality [36**••**].

## Very Low Carbohydrate Ketogenic Diet (VLCKD)

Ketogenic diets have very low amounts of carbohydrates (20–50 g), which come mainly from non-starchy vegetables [37]. Ketosis due to fat lipolysis readily occurs when carbohydrate intake is reduced to less than 50 g/day [38]. VLCKD initially increases total energy expenditure in patients with T2D, but this effect wanes off over time [29]. As explained, people who use VLCKD for weight loss (due its diuretic effect which leads to rapid weight loss) usually have a feeling of satiety caused by ketones. The most common negative adverse effect of such diets is called “keto-flu” which tends to improve spontaneously in few days to weeks after being on such diet. It causes symptoms like lightheadedness, dizziness, fatigue, exercise intolerance, lack of sleep, and constipation. [37]. Adherence to VLCKD diets is challenging, and their long-term effects are still lacking in the literature [39].

## Low-Fat–High-Carbohydrate Diet

Several studies have analyzed the opposite theory to the LC diet, focusing on increasing the amount or percentage of carbohydrates in diets (besides lowering the amount of fats or total caloric intake) and studying the effects of the HC diet on weight loss and glycemic control in patients with T2D. The macronutrient composition of such diets is regulated by the carbohydrate-to-fat (C/F) ratio. The benefit of increasing this ratio is not yet clearly determined since high C/F ratio may increase PPG, which in itself increases triglycerides and insulin secretion [40]. Evidence shows that high dietary carbohydrate intake elicits greater PPG responses compared to fats or proteins, both of which independently suppress this response [41].

The effects of replacing fats with carbohydrates among patients with T2D were evaluated in a meta-analysis [10]. Energy and protein levels among the included randomized trials were not largely different [10]. Also, no significant difference was identified between the two groups regarding A1C, fasting blood glucose (FBG), and cholesterol (total and LDL-cholesterol). However, other variables including fasting serum insulin, triglycerides, and HDL-cholesterol were mildly increased in patients who used low-fat–HC diets compared to those who consumed high-fat–LC diets [10]. Another meta-analysis showed inconsistent findings with no differences between low-fat–HC and high-fat–LC diets regarding their effects on glycemic control [23].

## Conclusion

Both low-carbohydrate and low-fat diets are effective in weight loss in patients with combined T2D and obesity, but low-carbohydrate diets are more effective on glycemic parameters, especially postprandial plasma glucose, glucose variability, serum triglycerides, and HDL-cholesterol. Low-carbohydrate diets are frequently associated with postprandial hyperlipidemia if dietary fat, instead of dietary protein, in these diets is mainly used to replace carbohydrates. Low-carbohydrate, high-protein diets are associated with increased satiety. Very low carbohydrate ketogenic diets reduce body weight and increase satiety due to ketosis, but long-term adherence is their major challenge. Literature is still lacking for well-designed RCTs to compare low-fat versus low-carbohydrate diets without confounding effects of from the behavioral aspects of eating. The ideal amount of carbohydrates, fats, and protein in an optimal diet for patients with a combination of obesity and T2D is still uncertain.
